# Responsible Translation of Stem Cell Research: An Assessment of Clinical Trial Registration and Publications

**DOI:** 10.1016/j.stemcr.2017.03.013

**Published:** 2017-04-13

**Authors:** Moses Fung, Yan Yuan, Harold Atkins, Qian Shi, Tania Bubela

**Affiliations:** 1 School of Public Health, Edmonton Clinic Health Academy, University of Alberta, Edmonton, AB T6G 1C9, Canada; 2 Faculty of Medicine & Dentistry, University of Alberta, Edmonton, AB T6G 2R7, Canada; 3 Ottawa Hospital Research Institute, University of Ottawa, Ottawa, ON K1H 8L6, Canada

**Keywords:** stem cell, clinical trial, clinical translation, clinical trial registry, clinical trial publication, stem cell tourism

## Abstract

We assessed the extent to which the publication of clinical trial results of innovative cell-based interventions reflects International Society for Stem Cell Research best practice guidelines. We assessed: (1) characteristics and time to publication of completed trials; (2) quality of reported trials; and (3) results of published trials. We identified and analyzed publications from 1,052 novel stem cell clinical trials: 179 (45.4%) of 393 completed trials had published results; 48 trials were registered by known stem cell tourism clinics, none of which reported results. Completed non-industry-sponsored trials initially published more rapidly, but differences with industry-sponsored trials decreased over time. Most publications reported safety, and 67.3% (mainly early-stage trials) reported positive outcomes. A higher proportion of industry trials reported positive efficacy. Heightened patient expectations for stem cell therapies give rise to ethical obligations for the transparent conduct of clinical trials. Reporting guidelines need to be developed that are specific to early-phase clinical trials.

## Highlights

• We assessed clinical trial publications of innovative cell-based interventions
• Only 54.5% of completed trials reported results
• Private clinics known to provide unproven stem cell therapies register “trials”
• Trial reporting guidelines are needed, specific to early-phase clinical trials

## Teaser

Fung and colleagues assessed publication patterns of clinical trials of novel stem cell therapies. Trial results remain underreported and stem cell tourism clinics are promoting their clinic activities on trial registries. Clinical trial reporting guidelines specific to early-phase clinical trials are needed.

## Introduction

The therapeutic promise of stem cell interventions has led to substantial international investment in research and clinical translation (Aging Analytics Agency, 2014, Caulfield et al., 2010). While most clinical trials of stem cell interventions remain focused on malignant and benign hematopoietic disorders, for which stem cell transplantation has been the standard of care for decades (Rettig et al., 2007), innovative but as yet unproven therapies are in clinical development (Heathman et al., 2015, Li et al., 2014, Trounson and McDonald, 2015), and a small number have received regulatory approval. Advances in stem cell research have raised the expectations of policy makers, funders, patients, and the public, but there is a large gap between expectations and clinical realities (Bubela et al., 2012). The public expects regenerative and possibly curative therapies for neurological conditions and injuries, heart disease, and autoimmune disorders (Bubela et al., 2012). High expectations combined with a lengthy trajectory of clinical development have created the environment for an expanding industry of clinics that provide unproven and questionable stem cell therapies (Charo, 2016, Master et al., 2014, Master and Resnik, 2011, Levine, 2010, Lau et al., 2008). Regulators and the research community are concerned about the rise in stem cell tourism, driven in part by patient anecdotes and high-profile/celebrity treatment profiles. Clinics exist in countries with lower regulatory standards and, in the United States, exploit regulatory loopholes to treat a wide range of conditions via the autologous administration of cell-based products (e.g., adipose-derived stem cells) (Turner and Knoepfler, 2016). Evidence suggests these clinics may harm patients; the *New England Journal of Medicine* reported on a case of glioproliferative lesion of the spinal cord that originated from infusions of “mesenchymal, embryonic, and fetal neural cells” at clinics in China, Argentina, and Mexico (Berkowitz, et al., 2016) and on three patients with age-related macular degeneration (AMD) who experienced severe bilateral vision loss after receiving autologous adipose-derived stem cell intravitreal injections at a clinic in Florida, an activity that does not require an investigational new drug application to the US Food and Drug Administration (Kuriyan et al., 2017).

While the toxicity profile is well understood for many hematopoietic stem cell interventions, long-term safety concerns persist for other cell types (Goldring et al., 2011, von Tigerstrom, 2008). The same properties that potentially make stem cells therapeutically valuable, such as proliferation, differentiation, migration/homing, and paracrine activity, make them potentially harmful (Sipp and Turner, 2012). Some cells, unlike a biologic or a small-molecule drug, are not metabolized and excreted from the body but are integrated into host tissue; it is conceivable, therefore, that safety issues may not become apparent for decades (Chapman and Scala, 2012, Dlouhy et al., 2014). Elevated risks give rise to expectations for precautionary measures that should be exercised in the design of early-stage clinical trials (Committee for Medicinal Products for Human Use, 2007), but trends in some countries are moving in the reverse direction. Researchers around the world are watching legislative reforms in Japan where regenerative medicine is a national priority. Japan has lowered the regulatory bar for regenerative medicine products by enabling up to 7 years of market approval, with concomitant reimbursement by the Japanese health system, based on data from early-stage trials that demonstrate safety and are “likely to predict efficacy” (Sipp, 2015).

Acknowledging these and other concerns, in 2016 the International Society for Stem Cell Research (ISSCR) updated its 2006 *Guidelines for the Conduct of Human Embryonic Stem Cell Research* and its 2008 *Guidelines for Clinical Translation of Stem Cells* to develop a comprehensive guidance document for stem cell research and clinical translation (International Society for Stem Cell Research, 2016). The 2016 Guidelines “bring all guidance together under common principles of research integrity, patient welfare, respect for research subjects, transparency, and social justice” (International Society for Stem Cell Research, 2016). Both the 2008 Guidelines and the 2016 *Guidelines for Stem Cell Research and Clinical Translation* identify the need to enhance transparency of clinical research as innovative cell-based interventions advance into clinical trials (International Society for Stem Cell Research, 2008, International Society for Stem Cell Research, 2016; Recommendation 3.3.6). The guidelines strongly encourage the publication of both positive and negative results and adverse events to ensure development of clinically effective and competitive stem cell-based interventions and to prevent participants in future clinical trials from being subjected to unnecessary risk (Kimmelman et al., 2016a, Kimmelman et al., 2016b, Daley et al., 2016, Caulfield et al., 2016, Isasi, 2009, International Society for Stem Cell Research, 2009). In addressing heightened public expectations and the rise of stem cell tourism, ISSCR recommends that researchers should present their results in peer-reviewed or professional scientific venues prior to communications with the lay media or patient advocacy groups and associations (Kimmelman et al., 2016a, Kimmelman et al., 2016b). Given that most stem cell clinical trials are early phase (Li et al., 2014), the Guidelines recommend that “researchers should take measures to maximize the scientific value of early-phase trials,” including the publication of “trials, methods, and sub-analyses in full” (International Society for Stem Cell Research, 2016; Recommendation 3.3.3.3). The Guidelines are concordant with the *NIH Policy on the Dissemination of NIH-Funded Clinical Trial Information* (NOT-OD-16-149), effective January 2017 which, in addition to requiring the registration of NIH-funded clinical trials, requires the submission of summary results as a condition of funding awards.

It is timely, therefore, to assess the extent to which the publication of clinical trial results of innovative cell-based interventions reflects ISSCR best practice guidelines. In the context of clinical trials for novel stem cell interventions, the objectives of our retrospective cohort study were to assess: (1) the characteristics and time to publication of completed trials; (2) the quality of reported trials against objective guidelines for trial conduct; and (3) the results of published trials. We included trial status as a variable in our assessment of the latter two objectives because, in our view, a trend toward premature reporting of results—often on a subset of participants prior to the completion of a trial—has the potential to contribute to heightened expectations for the field of regenerative medicine.

To our knowledge, this is the first study to provide a comprehensive assessment of the publication of clinical trials in the field of regenerative medicine. We did not, however, assess whether clinical trial results were presented at other professional venues.

## Results

### Descriptive

Of 1,052 novel trials in our dataset, 393 were completed, 81 were terminated or suspended, 22 were withdrawn, and the remaining 556 were in progress, including trials with unknown status. Of the trials completed, 179 (45.4%) had published results in 205 associated publications with English-language abstracts (Table 1). In September 2008, ClinicalTrials.gov developed the capacity to store trial results in conjunction with study protocol information (National Institutes of Health, 2015), but only 37 (3.5%) trials reported results in the registry even though 37.4% were listed as completed. However, 74.2% of all 357 publications identified the clinical trial registration number.

We identified 48 clinical trials with registration numbers on both ClinicalTrials.gov and the International Clinical Trials Registry Platform (ICTRP) from known clinics in North America, Eastern Europe, and Asia that offer unproven stem cell therapies (Table S1). Trials of adipose-derived stem cells or umbilical cord mesenchymal stem cells predominated for a range of conditions in both adult and pediatric participants. Most were recruiting or “enrolling by invitation.” None reported results.

### Publication of Results

Our analysis of the publication of results was based on 326 completed trials that met all inclusion criteria (PRISMA flow diagram, Figure S1A). Only one cell type (mononuclear fraction hazard ratio: 2.35; 95% CI: 1.32, 4.21) and industry sponsorship were associated with time to publication (Table S2). Industry sponsorship had a time-varying effect on time to publication (p = 0.004). Non-industry-sponsored trials initially published more rapidly after the end of the trial, but as time elapsed these differences decreased (hazard ratios were estimated to be 4.00, 1.93, 1.00, and 0.59 at 6 months, 1 year, 1.83 years, and 3 years, respectively). The median follow-up time from the end of trial data to the study end date (July 2015) for all completed trials was 4.2 years, where 77.1% trials in the analysis were followed up longer than 2.5 years. The Kaplan-Meier estimate of publication probability is 34.4% within 2.5 years.

### Quality and Outcomes of Published Clinical Trials

Our qualitative analysis of outcomes of all published clinical trials was based on 333 publications (Table 2), and our quantitative analyses were based on 297 publications that met additional inclusion criteria (PRISMA flow diagram, Figure S1B). The majority of published trials (88.8%) reported safety of the intervention without severe adverse events. Even though the majority of trials were early phase and focused on safety, 67.3% reported positive outcomes, i.e., incremental improvements in outcomes or positive results (Table 2); and 21.0% of trials reported null results, i.e., no improved outcomes. The majority of publications (76.9%) called for further studies, while 21.0% did not explicitly advocate for further studies. No trial characteristic had a significant overall effect on the publication of positive results, although phase III and phase III/IV trials were less likely to report positive results than phase I and phase I/II trials (odds ratio [OR] 0.11; p < 0.004).

We identified a number of problematic categories reported in publications (Table 3), including trial phase, sample size estimation, harms and their severity, and limitations. Journal impact factor (IF), trial status, graft type, cell type, and type of sponsor all had a significant effect on the completeness score of published results (Table S3). There were no differences in completeness of reporting between disease categories, and there was no improvement in reporting over time. Completeness score increased with percentile of journal IFs, meaning that higher impact journals reported more complete results (Table S3).

Of the 882 trials included in our statistical analyses, one-fourth (221; 25.1%) had at least one industry sponsor. Trials sponsored by public sector/research institutions had lower mean completeness scores than trials sponsored by industry (estimate: −1.51; p < 0.003). However, most published trials were publicly funded (228; 77.8%); the results of only 65 industry-sponsored trials were published in 67 publications. Safety was reported by 91.2% of publicly funded and 93.0% of industry-funded trials; a higher proportion of industry-funded than publicly funded trials reported positive efficacy (77.2% versus 67.2%); fewer industry-funded than publicly funded trials reported no efficacy (14.0% versus 22.7%); more publications advocating for further or continuing studies reported on industry-funded compared with publicly funded trials (82.4% versus 75.6%).

## Discussion

### Publication Rates

Underreporting of clinical trial results, especially phase I, remains a problem in many fields (Camacho et al., 2005, Decullier et al., 2009). For clinical trials of novel stem cell interventions, a publication rate of 45.5% for completed trials is consistent with other studies of publication rates. However, it remains problematic because the stem cell field combines high patient expectations, patient advocacy, strong political support, and therapeutic promise with regulatory concerns over safety and limited evidence of efficacy (Trounson and McDonald, 2015). The research community, research ethics boards, and regulators share responsibility to protect clinical trial participants from the undue risks associated with unproven interventions (Anderson and Kimmelman, 2014, Kimmelman, 2015). As costs of clinical research and development are high, obligations arise to public and private funders not to duplicate research efforts. The research community therefore fails patients and funders by not contributing scientific knowledge in high-risk, but potentially high-reward fields. Experimental stem cell therapies will not clear regulatory and reimbursement thresholds to become adopted therapies for the benefit of patients without an evidence base.

Unlike randomized controlled trials, early-stage clinical trials are not subject to mandatory reporting requirements, but should nevertheless publish results. Previous studies have reported publication rates of 56.5% for 4,347 interventional trials from academic centers, with 28.6% reporting within 24 months of study completion (Chen et al., 2016); 52% for a cohort of clinical studies approved by the Research Ethics Committee of the University of Freiburg, Germany (Blumle et al., 2014); and 48.4% for drug-evaluating clinical trials approved by the Ethics Committee of Hospital Vall d’Hebron in Barcelona, Spain, with results available for 68.9% (Blumle et al., 2014, Suñé et al., 2013). For later-stage trials, one study found that 29% of 585 large randomized registered trials remained unpublished with a median time from study completion to final literature search of 60 months (Jones et al., 2013). Large multicenter trials (77%) (von Elm et al., 2008) and trials for drugs that received regulatory licensure were more likely to be published (75%) than trials for drugs that stalled in clinical development (37%) regardless of disease type, sponsorship, trial phase, or geography (Hakala et al., 2015).

Other studies show differences in publication rates of trials sponsored by industry and academic centers. One study found that non-publication of results was more common for industry-funded trials (32%) than non-industry-funded trials (18%) (Jones et al., 2013); a second study reported that 40% of industry and 56% of non-industry-sponsored trials were published from a random sample of trials registered in ClinicalTrials.gov (Ross et al., 2012). One study concluded that nearly 300,000 study participants were exposed to the risks of clinical trials without benefit to society from the dissemination of results (Jones et al., 2013). Our results probably reflect the small number of industry-sponsored trials in our sample; these trials were less complete in their reporting of results and more likely to report positive results, including efficacy.

Clinical trials subject to Food and Drug Administration rules for mandatory reporting in clinical trial registries such as ClinicalTrials.gov include those for a drug, device, or biological agent having at least one United States site and in phase II or later. Such reporting has the potential to improve the transparency of clinical trials, and reduce publication bias and selective reporting (Viergever and Ghersi, 2011). However, one study found that only 22% of trials subject to mandatory reporting had reported within 1 year of study completion (Prayle et al., 2012). A second study found that of 8,907 interventional phase II or higher clinical trials completed between 2006 and 2009, 27.7% had a link to a published article, 26.6% deposited a summary of results in a registry, and only 9.2% had both; however, 54.9% had no evidence of results linkage between ClinicalTrials.gov and PubMed (Huser and Cimino, 2013). Furthermore, 78% of unpublished randomized clinical trials did not report results in the trial registry (Jones et al., 2013). In comparison, only 32.1% of our trials fell within this category, and of these, 29.9% had reported results in a clinical trial registry.

### Publication Bias

Numerous studies have identified a bias toward the publication of positive clinical trial results (Dwan et al., 2014, Hopewell et al., 2009, Vawdrey and Hripcsak, 2013). One study, comparing results of published versus unpublished clinical trials, found publication rates for positive results of 74% and 43%, respectively. While 57% of unpublished results showed no impact of an intervention, only 21% of published trials disclosed neutral outcomes, and 4% were negative or harmful. Not only are positive findings more likely to be published, they are also published more quickly than negative and null findings (Suñé et al., 2013, Hopewell et al., 2009), but positive outcomes are not associated with journal IF (Suñé et al., 2013). One study found an 89.4% publication rate for studies with positive results, compared with a 68.9% publication rate for studies with negative or null results that compared intervention with control (Suñé et al., 2013).

Our result of 67.3% publications reporting positive outcomes is concerning when combined with the early stage of most, and incomplete status of many, novel stem cell clinical trials. The majority of clinical trials in our study were focused on the safety of the intervention. However, one review article highlights publication bias in a late-stage stem cell therapy clinical trial (Galipeau, 2013). The negative phase III trial results for Prochymal (NCT00366145) were not published. Instead, in 2009 and 2010, the results were presented by the company as positive in press releases and an abstract at the 2010 American Society for Blood and Marrow Transplantation Tandem Meeting: “Our results suggest that the addition of Prochymal produced significant improvement without additive toxicity in patients with SR-GVHD involving visceral organs” (Martin et al., 2010).

### Quality of Clinical Trial Publications

Other studies have similarly found problematic areas for publication of clinical trial results. One study assessed the completeness and changes in registered data and reporting bias of randomized controlled trials in International Committee of Medical Journal Editors (ICMJE) journals after the implementation of the policy for trial registration (Huić et al., 2011). The study evaluated the completeness of nine items from the World Health Organization (WHO) 20-item Minimum DataSet relevant for the assessment of trial quality (World Health Organization, 2016). At the time of registration, the most commonly missing fields were key secondary outcomes (44.1%) and the primary outcome (38.8%) (Huić et al., 2011). Registration data and published data often differed (Huić et al., 2011). In addition to outcomes reporting, our study identified trial phase, sample size, harms and their severity, and limitations as areas of concern.

### Study Limitations

Our study has several limitations. First, there are inherent limitations in clinical trial registrations; the status of trials may not be updated, not all trials are registered, and not all registries have equivalent search capabilities with respect to text fields or indexing of keywords. Therefore, trials registered in ClinicalTrials.gov, the world's most comprehensive registry with the most powerful search capabilities, dominated our clinical trials dataset. Second, given the variability in completeness of the registry record, we may not have been able to locate trial publications because of missing data fields for principal investigator and other trial descriptors. Third, while we based our search of publications on the global landscape of clinical trials, we searched only for publications with English-language abstracts. Some clinical trials may have been published in other languages, especially those in Japan, China, and Iran. Finally, we allowed for a maximum of 2.5 years between the last clinical trials in our dataset (up to the end of 2012) and our publication searches in July 2015. Some trials may take longer to publish or have been prepared for publication but rejected by reviewers or journal editors. However, our longest time from completion of the trial to the last search date was 10.6 years for published trials and 15 years for unpublished trials.

### Ethics and Policy Implications

The field of stem cell research is dependent on public support and patient trust. Knowledge of the lack of reporting of clinical trial results in registries such as ClinicalTrials.gov is important for assessments of clinical and scientific validity, and, of equal importance, patients, family members, and friends are the largest user group of ClinicalTrials.gov (42% of use) (Zarin et al., 2013). This level of use by lay audiences is of concern in the context of stem cell research. It is evident that clinics engaged in stem cell tourism are registering studies to gain legitimacy and visibility. Indeed, such purported studies may harm patients. As described in the introduction, the *New England Journal of Medicine* reported on three women with AMD who were rendered legally blind after participating in what they believed to be a registered stem cell clinical trial (NCT02024269) (Kuriyan et al., 2017). The women “paid [$5,000 USD] for a procedure that had never been studied in a clinical trial, lacked sufficient safety data, and was performed in both eyes at the same time,” a practice that is “both atypical and unsafe” (Kuriyan et al., 2017). Such cases suggest that, at a minimum, regulatory loopholes that enable clinics to “treat” patients with “minimally manipulated” autologous cells for “homologous use” should be closed (Turner and Knoepfler, 2016).

In addition, however, information directed to patients considering enrollment in clinical trials or seeking treatment overseas, such as those promulgated by the ISSCR and the California Institute for Regenerative Medicine, should continue to warn patients that registration is not a mark of regulatory approval or quality control. The ISSCR, in particular, hosts a web resource (www.closerlookatstemcells.org) that features its *Patient Handbook on Stem Cell Therapies*, available in ten languages (International Society for Stem Cell Research, 2008), and other patient-oriented resources. The *Handbook* and the web resource provide information about stem cells and what questions to ask when considering stem cell treatments or enrollment in clinical trials.

Heightened patient expectations for stem cell therapies give rise to ethical obligations of researchers for the transparent conduct of clinical trials, as recommended by the ISSCR. Efforts of organizations such as the WHO to bring about comprehensive registration of clinical trials globally and the ICMJE requirements for registration prior to publication aim to increase public trust in biomedical research (Laine et al., 2007). ICH E3 CONSORT guidelines only recommend and do not prescribe reporting requirements, and published studies indicate that there is limited enforcement. Furthermore, publication guidelines need to be developed that are specific to early-phase clinical trials (the majority of trials for experimental stem cell therapies) and animate the 2016 ISSCR Guidelines, including adherence to primary endpoints and specific issues relevant to potential cell therapies, such as cell processing methods and dosing.

Guidance documents do not establish legally enforceable responsibilities (US Department of Health and Human Services, 2013). Nevertheless, it is evident from our data that enforcement by journals of reference to clinical trial registration is more effective than reporting of results in clinical trial registries. Clinical trial registries, therefore, need appropriate support for their efforts to monitor the status of ongoing clinical trials and the reporting of results to inform patients and decision makers of the development status of novel fields of biomedicine.

## Experimental Procedures

### Stem Cell Clinical Trial Sample

In our 2014 study (Li et al., 2014), we searched the term “stem cell” in ClinicalTrials.gov (which automatically includes all related terms, including “blast cell,” “cell progenitors,” “cells precursor,” “cells stems,” “hematopoietic progenitor cells,” “hemocytoblasts,” “precursor cell,” and “progenitor cell”) and “stem cell^∗^ NOT NCT0^∗^” in the WHO ICTRP (Li et al., 2014). After removing duplicate entries, we developed and applied criteria to identify clinical trials testing novel therapeutic applications of stem cells, which were applied by two independent coders; discrepancies were resolved by discussion with H.A. We included trials that: (1) used cells to stimulate non-hematopoietic organ regeneration; (2) used agents to stimulate stem or progenitor cell action for regenerative or therapeutic purposes, which excluded agents that targeted cancer stem cells; (3) used novel agents or processes for stem or progenitor cell mobilization for therapeutic purposes; and (4) used gene therapy or other ex vivo modified stem or progenitor cells. We excluded trials that: (1) were observational; (2) involved an established stem cell therapy for an established indication, namely hematopoietic stem cell therapies for hematopoietic cancers and anemias; and (3) investigated supportive measures for a stem cell therapy. Other reviews of the state of clinical translation in regenerative medicine have similarly excluded hematopoietic stem cell therapies (including bone marrow transplantation) for hematological cancers because these therapies are well established (Trounson and McDonald, 2015). Of the total 4,749 trials registered from 1988 (earliest record) to 1 January 2013, 1,052 unique trials met our inclusion criteria (Li et al., 2014). A similar number of cell-based clinical trials (1,342) met the inclusion criteria in Heathman et al. (2015). Our dataset, which includes novel clinical trials up to the end of 2012, provides a minimum 2.5-year time lag from registration to identify publication trends to July 2015.

### Publication Searches

We identified publications associated with each of the 1,052 trials by searching: (1) the ClinicalTrials.gov entries for published results; (2) PubMed, Embase, and Google Scholar for each registry number (e.g., NCT#); (3) those databases for the principal investigators named responsible for the trial in the registry; and (4) those databases for combinations of keywords from the official title of the trial listed in the registry. We developed our search strategy in consultation with a reference librarian at the University of Alberta and updated all searches to July 2015. We excluded 34 publications that were abstracts only, were review articles, or only reported preclinical data, leaving 357 publications with English-language abstracts (Figure S1B). We further excluded 24 publications that did not report the trial's primary outcomes, leaving a total of 333 publications (Figure S1B).

### Data Extraction

The ISSCR Guidelines call for adherence to standardized reporting guidelines for stem cell clinical trials, but adaptation of existing guidelines is necessary to account for the early phase of most stem cell clinical trials. We therefore developed and applied coding categories adapted from CONSORT (2010) (Moher et al., 2010, Schulz et al., 2010) and ICH E3 (Current Step 4 version) (ICH Expert Working Group No. E3, 1995) to assess the quality of reporting of trial results in publications (Table S4). The objective of these guidelines is to improve the reporting of randomized trials, which can be subject to bias if they lack methodological rigor (Jüni et al., 2001, Health Canada, 1996).

For each of our adapted coding categories, we recorded whether the information was (1) completely reported, (2) partially reported, (3) not reported, or (4) not applicable. We further applied qualitative criteria to assess statements on safety, efficacy, and recommendations for continuance in the published trials (Table S5). Two coders (M.F. and K. Yu) independently coded each article with good agreement for all variables (all kappas >0.83). Upon completion of the coding, where disagreement existed, the coders discussed the difference and came to a consensus for the final dataset. We calculated a completeness score for each publication, which summed the categories coded as “Completely Recorded.” The maximum achievable score was 23, which corresponded to the number of categories. Finally, we searched our dataset for the names of clinics that provide unproven stem cell therapies identified from the stem cell tourism literature (Li et al., 2014, Master et al., 2014, Master and Resnik, 2011, Levine, 2010, Lau et al., 2008, Turner and Knoepfler, 2016, Goldring et al., 2011, von Tigerstrom, 2008, Sipp and Turner, 2012, Ogbogu et al., 2013).

### Statistical Analysis

For statistical analyses, we excluded trials with the following status: enroll by invitation; not yet recruiting; pending; suspended; terminated; or withdrawn (n = 139), or that did not specify a graft type (n = 31), leaving a total of 882 trials. Of these 882 trials, 262 had 307 associated publications with English-language abstracts and 382 were completed (see Figure S1 for PRISMA flow diagrams). Statistical analyses, using R 3.2.1 (R Foundation for Statistical Computing, 2008), determined which trial characteristics (industry sponsor, trial phase, cell type used, graft type, and location of trial in a medium-high or very high human development country) were associated with: (1) the time to publication of completed trials; (2) the odds of reporting positive results; and (3) the completeness score. We also included trial status as a variable in analyses 2 and 3.

First, we used the Cox proportional hazards model to analyze the time from the end date of the trial to publication. We therefore excluded 14 trials with missing end dates and 41 trials that were published prior to but not after their end date. For the remaining 327 trials, one trial used both autologous and allogeneic graft types and was excluded due to the small sample size in that categorical level. We used the earliest publication date for trials with multiple publications. We examined the proportional hazards (PH) assumption for each trial characteristic. Industry involvement had a time-varying effect on the time to publication, thus we relaxed the PH assumption accordingly (Table S2). We used the following simple functional form to model the effect of industry sponsorship, while holding other variables constant:

$$HR\left(t\right)=\frac{h\left(t\;\text{|}\mathrm{public}\;\mathrm{funded}\;\mathrm{trials}\right)}{\;h\left(t|\mathrm{industry}\;\mathrm{sponsored}\;\mathrm{trials}\right)}\;=e^{\beta +\beta *\ln\left(t\right)}$$

where *h*(.) denotes the hazard function.

Second, we assessed the characteristics associated with the reporting of positive results for all trials. Because some trials resulted in more than one publication and one publication reported primary outcomes from two trials, we used the generalized estimating equation (GEE) method to account for these correlated observations and estimated adjusted ORs for each characteristic (Ziegler et al., 1998). We excluded the two publications from trials that used both autologous and allogeneic graft types because their inclusion resulted in unstable estimates of OR for graft type.

Finally, for the analysis of completeness of reporting, we excluded an additional ten publications where full text was not available in English. Most trials in our dataset would at most have a completeness score of 21 out of 23 because randomization and blinding are not used in early-phase non-randomized trials or trials without a control arm. When evaluating the remaining 297 full-text publications, we added journal IF and year of publication as explanatory variables. We used 2010, the year of publication of the most recent CONSORT guidelines, to create two publication year categories (after 2000 and prior to or in 2010). Based on an exploratory analysis, we created three IF categories (<30^th^ percentile, 31^st^–70^th^ percentile, and >71^st^ percentile). We used GEE to account for correlation among publications that resulted from the same trial. We measured the effect of each characteristic using the estimated adjusted mean score difference.

For each analysis, we chose the characteristic with the most trials as the default reference level, with the exception of sponsor and graft type. We report overall p values for each characteristic and individual p values that compare the effect of each level with the reference level within each characteristic.

## Author Contributions

T.B. and H.A. conceived the idea for the article and led the preliminary study to identify all novel stem cell clinical trials. M.F. in consultation with T.B. and H.A. elaborated and implemented a search strategy for clinical trial publications. M.F. coded the clinical trial publications. M.F., T.B., and Y.Y. designed and M.F., Y.Y., and Q.S. analyzed the data. M.F., Y.Y., and T.B. wrote the article. All authors edited, reviewed, and approved the final version. T.B. is the guarantor of the study.

## Figures and Tables

**Table 1:** Characteristics of 1,052 Registered Stem Cell Clinical Trials and 286 Trials That Published Primary Outcome Data in 333 Publications

Characteristics	No. of Trials (n)	No. of Trials with At Least One Associated Publication^a^ n (%)
Status		
Completed	393	179 (45.5)
Active not recruiting	133	18 (13.5)
Expanded access no longer available	2	1 (50.0)
Recruitment ongoing	195	28 (14.4)
Unknown	190	43 (22.6)
Enrolling by invitation	17	1 (5.9)
Not yet recruiting, pending	19	1 (5.3)
Suspended, terminated, withdrawn	103	15 (14.6)
Phase		
Phase I, phase I & II	527	146 (27.7)
Phase II, phase II & III	265	72 (27.2)
Phase III, phase III & IV	44	11 (25.0)
Phase IV	19	13 (68.3)
Not applicable or phase 0	197	44 (22.3)
Graft type		
Allogeneic	305	62 (20.3)
Autologous	588	174 (29.6)
Autologous and allogeneic	8	2 (25.0)
No graft product	119	42 (35.3)
Unspecified	32	6 (18.8)
Cell type^b^		
Mesenchymal	403	87 (21.9)
CD34^+^ fraction	113	32 (28.3)
Endothelial progenitor	65	26 (40.0)
Hematopoietic	184	50 (27.2)
Mononuclear fraction	93	41 (44.1)
Combined	33	13 (39.4)
Others	161	37 (23.1)
Country^c^		
Very high human development	724	203 (28.0)
Medium-high human development	328	83 (25.3)
Funding		
Public	790	221 (30.0)
Industry	262	65 (24.8)
Overall	1052	286 (27.2)

a Note that 38 clinical trials had more than one associated publication.

b Cell types: Hematopoietic cell refers to a graft collected directly from patients used without modification (other than possible cryopreservation and thawing). Mononuclear fraction refers to a hematopoietic cell product purified by density gradient separation or other means that removes granulocytes and red blood cells. CD34^+^ fraction refers to hematopoietic cell products that underwent purification using a monoclonal antibody to CD34. All these products contain hematopoietic stem cells (HSCs) and are used for hematopoietic engraftment. The other category includes: studies using neural precursors or stem cells (n = 24), CD133 purified HSC or angiogenic cell precursor populations (n = 22), limbal stem cells (n = 16), cell products derived from embryonic stem cell (n = 6), cardiac cells (n = 6), products used in four or fewer trials (n = 43), and trials in which the stem cell product was not specified (n = 20). Specific cell products used in <4 trials included: adrenocortical, bone progenitor, chondrocytes, fibroblasts or fibrocytes, germ cells, oral mucosa and dental cells, pancreatic islet precursors, placental skeletal muscle, and skin or hair follicle stem or precursor cell products.

c Countries were classified according to the United Nations Development Program: Human Development Index—Country Profiles (http://hdr.undp.org/en/countries).

**Table 2:** Characteristics of 333 Publications of Registered Stem Cell Clinical Trials that Reported Primary Outcomes Data

Clinical Trial Characteristic	No. of Publications (n)	No. of Publications with Positive Results n (%)	Completeness Score^a^ (mean ± SD)
Status			
Completed	210	139 (66.1)	16.6 ± 3.7
Active not recruiting	22	17 (77.3)	15.2 ± 3.8
Expanded access no longer available	2	2 (100.0)	15.5 ± 2.1
Recruitment ongoing	31	23 (74.2)	14.0 ± 4.1
Unknown	48	34 (70.8)	15.8 ± 3.1
Enrolling by invitation	1	1 (100.0)	13.0 ± 0.0
Not yet recruiting, pending	1	1 (100.0)	12.0 ± 0.0
Suspended, terminated, withdrawn	18	7 (38.9)	15.6 ± 3.1
Phase			
Phase I, phase I & II	167	125 (74.8)	15.5 ± 3.7
Phase II, phase II & III	93	59 (63.4)	16.8 ± 3.8
Phase III, phase III & IV	12	3 (25.0)	14.5 ± 2.9
Phase IV	14	8 (57.1)	16.1 ± 2.6
Not applicable or phase 0	47	29 (61.7)	16.9 ± 3.9
Graft type			
Allogeneic	69	47 (68.1)	15.4 ± 3.8
Autologous	211	149 (70.6)	16.2 ± 3.8
Autologous and allogeneic	2	2 (100.0)	20.5 ± 0.7
No graft product	45	22 (48.9)	16.2 ± 3.2
Unspecified	6	4 (66.7)	15.3 ± 3.1
Cell type^b^			
Mesenchymal	100	75 (75.0)	16.6 ± 3.3
CD34^+^ fraction	37	31 (81.6)	16.9 ± 3.6
Endothelial progenitor	28	19 (90.4)	15.1 ± 2.3
Hematopoietic	61	31 (50.8)	16.3 ± 4.0
Mononuclear fraction	52	29 (55.8)	15.1 ± 4.3
Combined	14	10 (71.4)	15.9 ± 3.9
Others	41	29 (70.7)	15.4 ± 4.1
Country			
Very high human development	245	160 (65.3)	16.3 ± 3.6
Medium-high human development	88	64 (72.7)	15.5 ± 3.9
Funding			
Public	263	173 (65.8)	15.7 ± 3.8
Industry	70	8 (72.9)	17.2 ± 3.3
Journal impact factor			
0–2.98	87	57 (65.5)	14.5 ± 3.3
2.98–3.88	139	91 (65.5)	16.3 ± 3.7
3.88–41.5	107	76 (71.0)	17.2 ± 3.8
Year of publication			
Prior to and during 2010	223	156 (69.9)	16.2 ± 3.8
After 2011	110	68 (61.8)	15.9 ± 3.7
Overall	333	224 (67.3)	16.0 ± 3.7

a Ten non-English-language publications were excluded from our analysis of completeness score.

b Cell types: hematopoietic cell refers to a graft collected directly from patients used without modification (other than possible cryopreservation and thawing). Mononuclear fraction refers to a hematopoietic cell product purified by density gradient separation or other means that removes granulocytes and red blood cells. CD34^+^ fraction refers to hematopoietic cell products that underwent purification using a monoclonal antibody to CD34. All these products contain HSCs and are used for hematopoietic engraftment. The other category includes: studies using neural precursors or stem cells (n = 24), CD133 purified HSC or angiogenic cell precursor populations (n = 22), limbal stem cells (n = 16), cell products derived from embryonic stem cell (n = 6), cardiac cells (n = 6), products used in four or fewer trials (n = 43), and trials in which the stem cell product was not specified (n = 20). Specific cell products used in <4 trials included: adrenocortical, bone progenitor, chondrocytes, fibroblasts or fibrocytes, germ cells, oral mucosa and dental cells, pancreatic islet precursors, placental skeletal muscle, and skin or hair follicle stem or precursor cell products.

**Table 3:** Completeness of Reporting in 23 Categories for 323^a^ English-Language Publications of Novel Stem Cell Clinical Trials

Reporting Category^b^	Completely Reported n (% of Applicable Trials)	Partially Reported n (% of Applicable Trials)	Not Reported n (% of Applicable Trials)	Category Not Applicable n
**Background**				

Background/rationale	310 (93.1)	12 (3.6)	1 (0.3)	0

**Methods**				

Interventions	310 (93.1)	12 (3.6)	1 (0.3)	0
Trial design	275 (82.6)	27 (8.1)	21 (6.3)	0
Primary outcome	266 (79.9)	54 (16.2)	3 (0.9)	0
Statistical methods	253 (76.0)	16 (4.8)	54 (16.2)	0
Participant criteria	246 (73.9)	57 (17.1)	19 (5.7)	0
Secondary outcome	201 (63.8)	43 (13.6)	71 (22.5)	8
Trial phase	183 (55.0)	1 (0.3)	139 (41.7)	0
Blinding	119 (55.6)	35 (16.4)	60 (28.0)	109
Randomization	116 (54.7)	54 (25.5)	42 (19.8)	111
Sample size	94 (28.2)	19 (5.7)	210 (63.1)	0

**Results**				

Primary endpoint	295 (88.6)	14 (4.2)	14 (4.2)	0
Harms/side effects	232 (69.7)	11 (3.3)	80 (24.0)	0
Participant flow	200 (60.1)	87 (26.1)	36 (10.8)	0
Deaths	190 (57.1)	2 (0.6)	131 (39.3)	0
Recruitment period	168 (50.5)	5 (1.5)	150 (45.0)	0
Severity of harms	153 (45.9)	16 (4.8)	154 (46.2)	0

**Conclusion**				

Generalizability	300 (90.1)	17 (5.1)	6 (1.8)	0
Interpretation	272 (81.7)	43 (12.9)	8 (2.4)	0
Limitations	191 (57.4)	66 (19.8)	66 (19.8)	0

**Other**				

Protocol	295 (88.6)	7 (2.1)	21 (6.3)	0
Funding	276 (82.9)	5 (1.5)	42 (12.6)	0
Registration	244 (73.3)	6 (1.8)	73 (21.9)	0

a Ten non-English-language publications were excluded from our analysis of completeness score. Percentages exclude trials with non-applicable reporting categories.

b Reporting categories modified for early-phase clinical trials from CONSORT (Moher et al., 2010, Schulz et al., 2010).

## Supplemental Information

Document S1. Figure S1 and Tables S1–S5

Document S2. Article plus Supplemental Information
