# Supplementary material for: Responsible Translation of Stem Cell Research: An Assessment of Clinical Trial Registration and Publications
Source: Stem Cell Reports. 2017 Apr 13;8(5):1190–201. doi: 10.1016/j.stemcr.2017.03.013 (PMC5425617; doi:10.1016/j.stemcr.2017.03.013)
Supplement: Document S1. Figure S1 and Tables S1–S5 [file mmc1.pdf]

Stem Cell Reports, Volume *8*

Supplemental Information

Responsible Translation of Stem Cell Research: An Assessment of Clinical Trial Registration and Publications

Moses Fung, Yan Yuan, Harold Atkins, Qian Shi, and Tania Bubela

Supplemental Materials for

Responsible translation of stem cell research: an assessment of clinical trial registration and publications

Authors: Moses Fung[1,2], Yan Yuan[1], Harold Atkins[3], Qian Shi[1], Tania Bubela[1*]

Affiliations:

[1] School of Public Health, Edmonton Clinic Health Academy; University of Alberta; Edmonton, Alberta, T6G 1C9; Canada

[2] Faculty of Medicine & Dentistry; University of Alberta; Edmonton, Alberta, T6G 2R7; Canada

[3] Ottawa Hospital Research Institute; University of Ottawa; Ottawa, Ontario, K1H 8L6; Canada

*Corresponding author. Email: tbubela@ualberta.ca

This document file includes:

Figs. S1 to S3

Tables S1 to S5

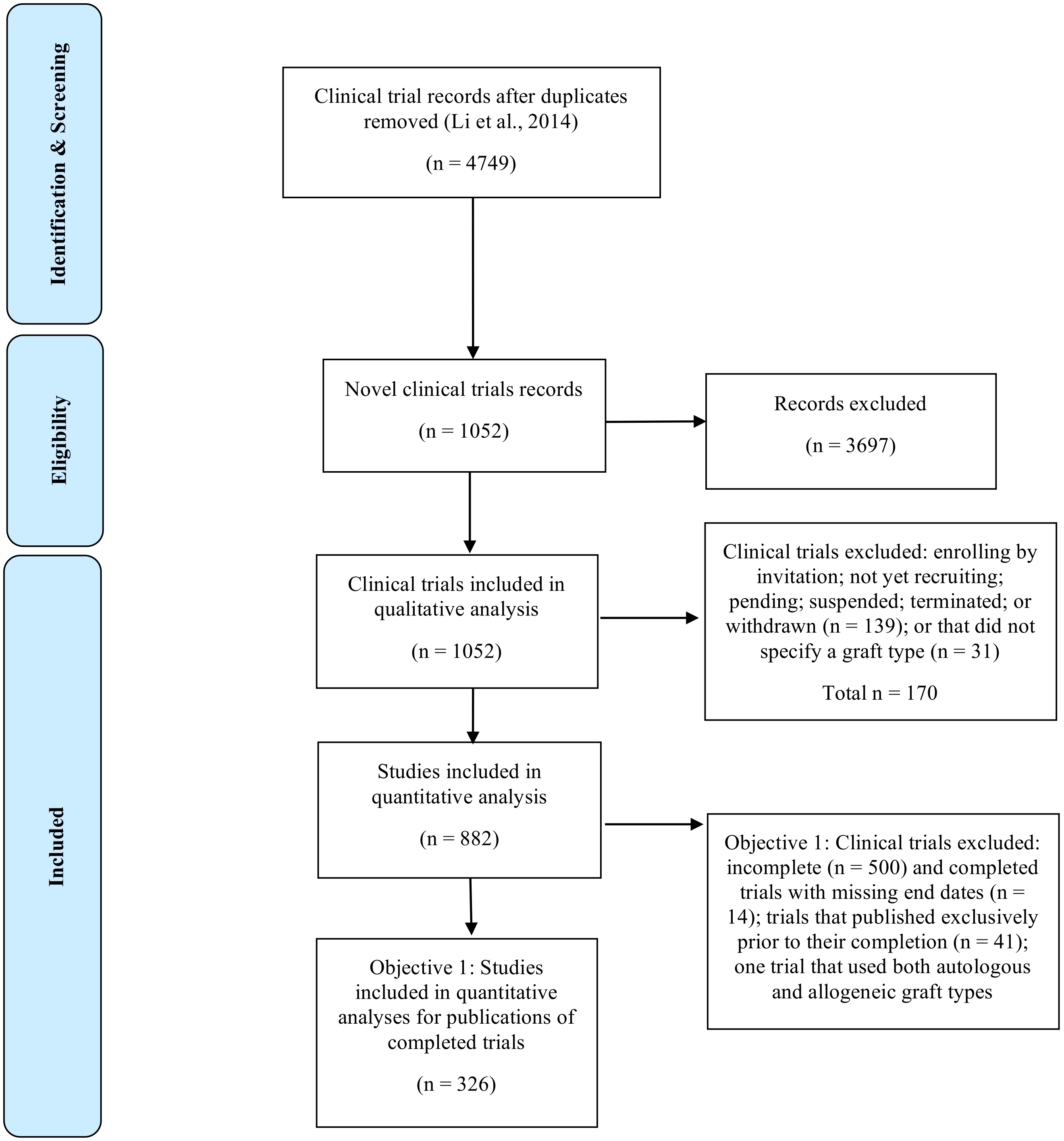

Fig. S1a.

Modified PRISMA Flow Diagram for Clinical Trials.

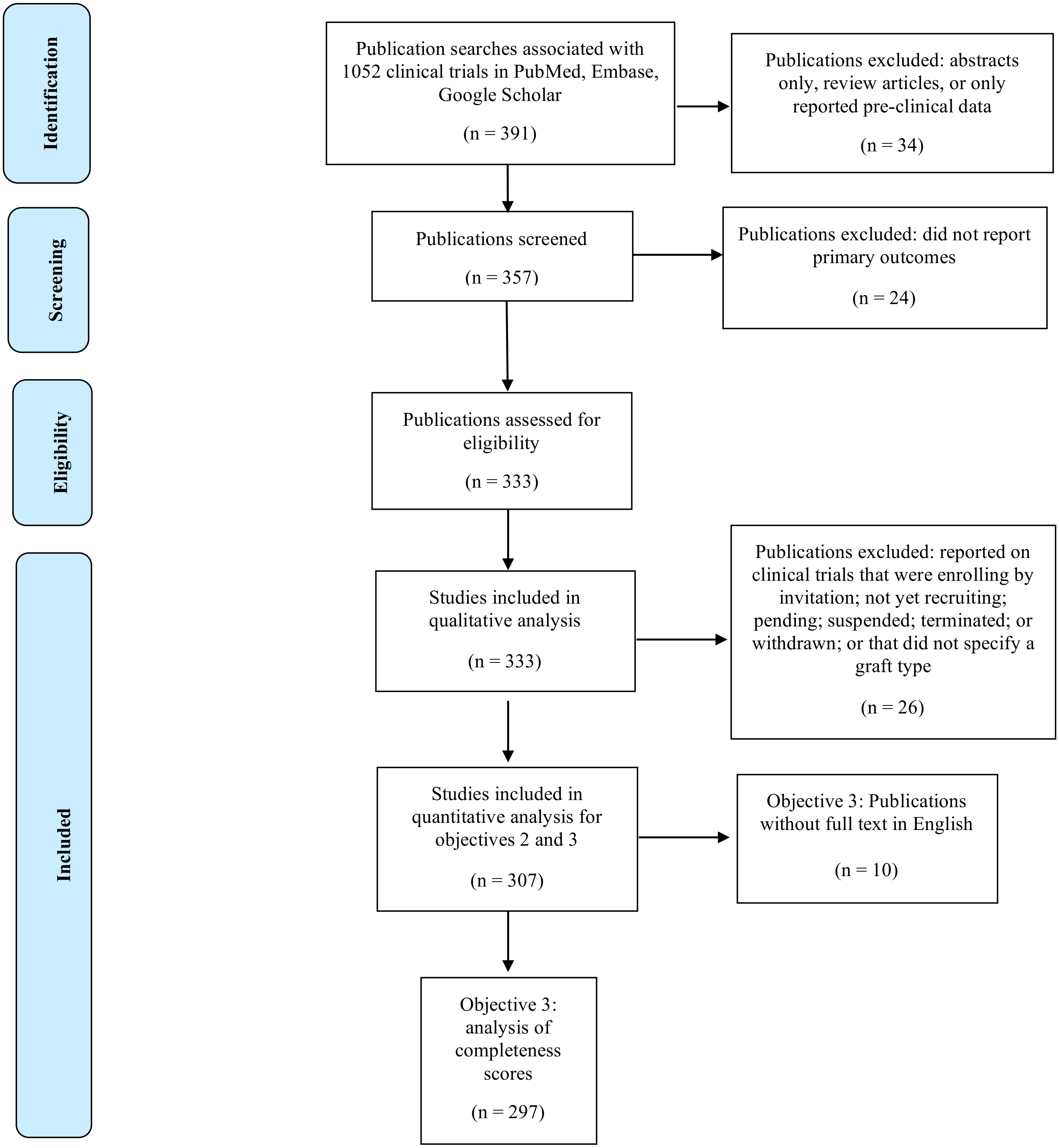

Fig. S1b.

Modified PRISMA Flow Diagram for Clinical Trial Publications.

Table S1. Characteristics of clinical trials registered by clinics that provide unproven cell therapies to patients. No results have been published.

Clinic Name	Country	Clinical trial number (Phase)	Participants (enrollment)	Intervention	Conditions	Start date
Adistem	Philippines	NCT00703599 (1/2)	30 (Child)	Autologous Adipose-derived Stem cells	Type I Diabetes	Nov-07
		NCT00703612 (1/2)	34 (Adult)		Type II Diabetes	Nov-07
Ageless Regenerative Institute	United States - Florida	NCT01453751 (1/2)	500 (Adult)	Adipose-Derived Stem Cell Therapy	Diabetes Mellitus Type II	Mar-14
		NCT01559051 (1/2)	200 (Adult)		Chronic Obstructive Pulmonary Disease	Mar-14
		NCT01739504 (1/2)	500 (Adult)		Osteoarthritis	Mar-14
		NCT02087397 (1/2)	500 (Adult)		Erectile Dysfunction	Mar-14
		NCT02099500 (1/2)	200 (Adult)		Critical Limb Ischemia	Mar-14
		NCT01453764 (1/2)	10 (Adult)		Multiple Sclerosis	Oct-14
		NCT01453777 (1/2)	10 (Adult)		Brain Lesion (General)	May-11
		NCT01453803 (1/2)	10 (Adult)		Parkinson's Disease	May-11
		NCT01453816 (1/2)	10 (Adult)		Renal Failure	May-11
		NCT01453829 (1/2)	10 (Adult)		Stroke	Oct-14
		NCT01501461 (1/2)	10 (Adult)		Frailty Syndrome	May-11
		NCT01502488 (1/2)	10 (Child)		Autism	Oct-14
		NCT01502501 (1/2)	10 (Adult)		Non-Ischemic Congestive Heart Failure	May-11
		NCT01502514 (1/2)	10 (Adult)		Ischemic Congestive Heart Failure	May-11
		NCT01974128 (1/2)	10 (Adult)		Acute Myocardial Infarction	Oct-14
Bieke Biotech	India	NCT00922389 (1/2)	36 (Adult)	Peripheral Blood Derived Mononuclear Cells	Diabetic Foot, Critical Limb Ischemia, Leg Ulcers	Jul-09
Shenzhen Beike Bio-Technology	China	NCT01539902 (2)	25 (Both)	Human Umbilical Cord Derived Mesenchymal Stem Cell	Lupus Nephritis	Feb-12
		NCT01742533 (1/2)	40 (Adult)		Premature Ovarian Failure	Mar-12
		NCT01343511 (1/2)	37 (Child)		Autism	Mar-09
		NCT01342250 (1/2)	20 (Adult)		Liver Cirrhosis	Oct-10
		NCT01360164 (1/2)	20 (Both)		Hereditary Ataxia	Jan-10
		NCT01364246 (1/2)	20 (Adult)		Progressive Multiple Sclerosis, Neuromyelitis	Jan-10
		NCT01443689 (1/2)	20 (Adult)		Optica	Jul-11
		NCT01610440 (1/2)	15 (Child)		Burns	Oct-11
					Duchenne Muscular Dystrophy	
Bioheart	United States - Florida	NCT00050765 (1)	15 (Adult)	Autologous Myoblasts (Myocell)	Congestive Heart Failure, Myocardial Infarction	Aug-06
		NCT00054678 (1)	20 (Adult)			Feb-03
		NCT00375817 (2)	40 (Adult)			Nov-05
		NCT00526253 (2/3)	170 (Adult)		Congestive Heart Failure, Myocardial Infarction	Sep-07

		NCT02024269 (?)<br>NCT02041000 (?)<br>NCT02097862 (?)	100 (Adult)<br>100 (Adult)<br>100 (Adult)	Adipose-derived stem cells	Dry Macular Degeneration<br>Chronic Obstructive Pulmonary Disease<br>Degenerative Disk Disease	Dec-13<br>Jan-14<br>Mar-14
Institute of Regenerative and Cellular Medicine	United States - California	NCT01947348 (?)<br>NCT01947348 (?)<br>NCT01947348 (?)	30 (Adult)<br>30 (Adult)<br>30 (Adult)	A3 SVF (Stromal Vascular Fraction)	Osteoarthtitis	Sep-12<br>Sep-12<br>Sep-12
NueroVita Clinic	Russia	NCT01759810 (2/3)<br>NCT01759810 (2/3)<br>NCT01782274 (2/3)<br>NCT01782274 (2/3)<br>NCT01782287 (2/3)<br>NCT01782287 (2/3)	60 (Adult)<br>60 (Adult)<br>60 (Adult)<br>60 (Adult)<br>60 (Adult)<br>60 (Adult)	Dendritic vaccine, allogeneic & autologous hematopoietic stem cells, cytotoxic lymphocytes	Glioblastoma<br>Glioblastoma<br>Neoplasm Metastasis<br>Neoplasm Metastasis<br>Neoplasm Metastasis<br>Neoplasm Metastasis	Dec-12<br>Dec-12<br>Dec-12<br>Dec-12<br>Dec-12<br>Dec-12
NuroGeneration	United States - California	NCT01329926 (0)	20 (Adult)	Adult Human-derived Neural Stem Cells	Parkinson’s Disease	Jun-11
TheraVitae	Canada, Israel, Thailand, Hong Kong	NCT00416663 (1/2)<br>NCT00523224 (1)<br>NCT00523731 (1)<br>NCT00384514 (2)	10 (Adult)<br>5 (Adult)<br>6 (Adult)<br>24 (Adult)	Angiogenic Cell Precursors (ACPs) or Vescell TM	Angina pectoris<br>Congestive Heart Failure<br>Peripheral Arterial Disease, Critical Limb Ischemia<br>Coronary Artery Disease	Aug-07<br>Sep-07<br>Mar-07<br>Sep-06
XCell-Center		EUCTR2010-024391-25-CZ (2)	88 (Adult)	ASCT01 (Bone Marrow Derived Stem Cells)	Critical limb Ischemia	May-11

Table S2. Characteristics of publications of 326 completed novel stem cell clinical trials associated with publication.

Characteristic	% Published (n)	Hazards Ratio	95% Confidence Interval for Hazard Ratio	p-value
**Sponsor**				
Industry (reference group)	41.05 (95)			
Public sector	36.80 (231)	1.93‡	(1.03,3.61)	**0.04**
**Phase**				
Phase I,Phase I Phase II (reference group)	40.39 (151)	1	-	
Phase II, Phase II Phase III	40.50 (79)	0.93	(0.60,1.45)	0.75
Phase III, Phase III Phase IV	25.00 (12)	0.49	(0.15,1.60)	0.24
Phase IV	58.33 (12)	1.98	(0.77,5.05)	0.15
N/A, Phase 0	29.17 (72)	0.75	(0.44,1.24)	0.27
**Graft type**				
Allogeneic (reference group)	29.76 (84)	1	-	
Autologous	43.82 (178)	1.42	(0.85,2.39)	0.19
No graft product	32.81 (64)	1.10	(0.51,2.38)	0.81
**Cell type** [a]				
Mesenchymal (reference group)	35.59 (118)	1	-	
CD34+ fraction	37.50 (24)	0.65	(0.30,1.40)	0.27
Endothelial progenitor	33.33 (30)	0.68	(0.25,1.86)	0.46
Hematopoietic	35.48 (62)	0.82	(0.45,1.49)	0.51
Mononuclear fraction	64.52 (31)	2.35	(1.32,4.21)	**0.004**
Combination	62.50 (16)	1.79	(0.88,3.67)	0.11
Others	24.44 (45)	0.64	(0.32,1.28)	0.21
**Country**[b]				
Medium-High Human Development (reference group)	36.90 (84)			
Very High Human Development	38.43 (242)	1.34	(0.87,2.09)	0.18

‡ The hazard ratio for Public sector vs. Industry sponsored trials is time varying. The number shown here is the estimated hazard ratio at 1 year (from the end of the trial).

[a] Cell types: Hematopoietic cell refers to a graft collected directly from patients used without modification (other than possible cryopreservation and thawing). Mononuclear fraction refers to a hematopoietic cell product purified by density gradient separation or other means that removes granulocytes and red blood cells. CD34+ fraction refers to hematopoietic cell products that underwent purification using a monoclonal antibody to CD34. All these products contain hematopoietic stem cells (HSC) and are used for hematopoietic engraftment. The other category includes: studies using neural precursors or stem cells (n=24), CD133 purified HSC or angiogenic cell precursor populations (n=22), limbal stem cells (n=16), cell products derived from embryonic stem cell (n=6), cardiac cells (n=6), products used in 4 or fewer trials (n=43), and trials in which the stem cell product was not specified (n=20). Specific cell products used in <4 trials included: adrenocortical, bone progenitor, chondrocytes, fibroblasts or fibrocytes, germ cells, oral mucosa and dental cells, pancreatic islet precursors, placental skeletal muscle, and skin or hair follicle stem or precursor cell products.

[b] Countries were classified according to the United Nations Development Programme: Human Development Index – Country Profiles (http://hdr.undp.org/en/countries)

Table S3. Characteristics of publications on novel stem cell clinical trials associated with the completeness score for reporting using the Generalized Estimating Equation (GEE) method.

Characteristic	Estimate	95% Confidence Interval
**Intercept**	15.76	14.27 to 17.24
**Journal Impact Factor (5 Year)**		
<30th percentile [0-2.977] (reference group)	0	-
30th-70th percentile [2.977-3.883]	1.76	0.76 to 2.76
> 70th percentile [3.883-41.541]	3.14	2.06 to 4.21
**Year of publication**		
After 2011 (reference group)	0	-
Before 2011	-0.02	-0.87 to 0.84
**Country**		
Medium-High Human Development Countries (reference group)	0	-
Very High Human Development Countries	-0.73	-1.69 to 0.23
**Clinical Trial Status**		
Completed, Active not recruiting etc. (reference group)	0	-
Recruitment ongoing	-2.59	-4.14 to -1.04
Unknown	-0.36	-1.38 to 0.66
**Sponsor**		
Industry (reference group)	0	-
Public sector	-1.51	-2.52 to -0.50
**Phase**		
Phase I, Phase I Phase II (reference group)	0	-
Phase II, Phase II Phase III	0.90	-0.07 to 1.88
Phase III, Phase III Phase IV	-1.06	-2.49 to 0.38
Phase IV	1.31	0.06 to 2.56
N/A, Phase 0	1.25	-0.49 to 3.00
**Graft type**		
Allogeneic (reference group)	0	-
Autologous	1.48	0.22 to 2.73
No graft product	2.02	0.28 to 3.76
**Cell type***		
Mesenchymal (reference group)	0	-
CD34+ fraction	-0.43	-1.72 to 0.87
Endothelial progenitor	-3.02	-4.49 to -1.54
Hematopoietic	-0.74	-2.06 to 0.57
Mononuclear fraction	-1.71	-3.12 to -0.29
Combination	-0.89	-2.74 to 0.96
Others	-1.56	-3.12 to 0.00

* Cell types: Hematopoietic cell refers to a graft collected directly from patients used without modification (other than possible cryopreservation and thawing). Mononuclear fraction refers to a hematopoietic cell product purified

by density gradient separation or other means that removes granulocytes and red blood cells. CD34+ fraction refers to hematopoietic cell products that underwent purification using a monoclonal antibody to CD34. All these products contain hematopoietic stem cells (HSC) and are used for hematopoietic engraftment. The other category includes: studies using neural precursors or stem cells (n=24), CD133 purified HSC or angiogenic cell precursor populations (n=22), limbal stem cells (n=16), cell products derived from embryonic stem cell (n=6), cardiac cells (n=6), products used in 4 or fewer trials (n=43), and trials in which the stem cell product was not specified (n =20). Specific cell products used in <4 trials included: adrenocortical, bone progenitor, chondrocytes, fibroblasts or fibrocytes, germ cells, oral mucosa and dental cells, pancreatic islet precursors, placental skeletal muscle, and skin or hair follicle stem or precursor cell products.

Table S4. Characteristics of reported criteria (background, methods and other) for published novel stem cell clinical trials adapted from CONSORT and ICH E3 Guidelines.

Criterion Adapted from CT Reporting Guidelines	Reported	Partially Reported	Not Reported
1. Background	Relevant background and rationale reported.	Background and rationale not clearly explained.	No information on background or rationale reported.
**Methods**			
2. Trial Phase	Phase of the trial or stage of research if pilot study reported.	Not Applicable.	Phase or stage of the trial not reported.
3. Trial Design	Description of trial design (e.g., parallel, factorial), including allocation ratio and important changes to methods after trial commencement, if necessary.	Incomplete description of trial design, including allocation ratio, or incomplete reporting of important changes to methods.	Trial design not reported.
4. Participant Criteria	Eligibility (inclusion and exclusion) criteria for participants, settings and locations of data collection reported.	Incomplete eligibility criteria for participants, or missing information on settings and locations of data collection.	Eligibility criteria for participants not reported.
5. Interventions	Interventions for each group reported with sufficient detail to allow replication, including how and when they were administered (or recorded as observational).	Interventions for each group partially reported without sufficient detail to allow replication, or lacking information on how and when interventions were administered.	No information on interventions reported.
6. Primary Outcome	Defines pre-specified primary outcome measure(s), including how and when they are assessed.	Defines primary and secondary outcome measures, but reports no information on how and when they are assessed.	No information on outcome measures reported.
7. Secondary Outcome	Defines pre-specified or ad-hoc secondary outcome measure(s), including how and when they are assessed.	Defines secondary outcome measures, but reports no information on how and when they are assessed.	No information on outcome measures reported.
8. Sample Size Estimation	Planned sample size method for its determination reported.	Planned sample size stated; method for determination of sample size not reported.	No information on sample size reported.
9. Randomization (Not applicable for non-randomized clinical trials)	Study identified as randomized, and reports type of randomization and method for random allocation.	Study identified as randomized but type of randomization or method for random allocation sequence not reported.	Study identified as randomized but no information on randomization reported.
10. Blinding (Not applicable for phase I and II trials without a randomized control arm)	Study identified as blinded, blinded parties identified, and methods of blinding reported (or	Study identified as blinded but blinded parties or method of blinding not reported.	Study identified as blinded but no information on blinding reported.

	study identified as open-labeled).		
11. Statistical Methods	Statistical methods reported for comparison of groups of primary and secondary outcomes.	Statistical methods used not explicitly reported.	No statistical methods reported.
**Results**			
12. Participant Flow	Reported number of participants assigned, treated and analysed, with diagram, including losses and exclusions.	Missing information on number of participants assigned, treated and analysed, or number of patients lost or excluded.	No information on participant flow reported.
13. Recruitment Period	Dates for recruitment and follow-up periods reported (month and year), including why trial stopped (if applicable).		Dates for recruitment and follow-up periods not reported.
14. Primary Endpoint	Number of patients with events and % of analysis data set per group reported.	Data insufficient to be included in meta-analysis.	Multiple primary endpoints, without appropriate adjusting for multiple testing, contradictory information concerning primary endpoint.
15. Harms/Side Effects	Important harms or unintended effects reported.		No information on harms or unintended effects reported.
16. Severity	Severity of harms reported in a table with a toxicity grade.		Severity of harms in a table with a toxicity grade not reported.
17. Deaths	Number of patients that died during the trial reported (if any).		Number of patients that died during the trial not reported.
18. Limitations	Trial limitations, addressing sources of potential bias, imprecision reported.	Trial limitations not explicitly addressed.	No limitations reported.
19. Generalizability	External validity and applicability of trial findings reported.	Generalizability of results not consistent with results, or not fully disclosed.	No inferences of generalizability reported.
20. Interpretation	Interpretation consistent with results, balancing benefits and harms and considering other relevant evidence.	Interpretation not consistent with results, or missing balance of benefits and harms or consideration of other relevant evidence.	Interpretation not available.
**Other**			
21. Registration	Registration number and name of trial registry reported.	Mentioned as registered, but either number or registry not reported.	No registration reported.
22. Availability of Full Protocol	Accessible link to full trial protocol available (supplementary documents, other site) or		Full trial protocol not accessible.

	protocol reported in the paper.		
23. Funding	Sources of funding and other support reported.		Sources of funding and other support not reported.

Table S5. Qualitative Criteria for Assessing the Safety and Efficacy of Reported Stem Cell Clinical Trials.

Qualitative Assessment of Clinical Trial Reports	Definition
1. Trial Status	In Progress: interim report published.<br>Completed: clinical trial completed with results analysed and presented.<br>Withdrawn: halted prematurely, prior to enrollment of first patient:<br>• Reasons given;<br>• Reasons not given.<br>Suspended: recruiting or enrolling has halted prematurely but potentially will resume.<br>Terminated: recruiting or enrolling has halted prematurely and will not resume; participants are no longer being examined or treated:<br>• Reasons given;<br>• Reasons not given.
2. Presence of Serious Side Effects	Side Effects Observed: organ system involved according to NCIC-CTY standard (e.g., hematopoietic, cardiac, gastrointestinal).<br>No Severe Side Effects Observed.<br>Unknown: Neither presence nor absence of side effects reported.
3. Reported Expectation of Efficacy	Positive: patient outcomes improved, results were statistically significant ($P < 0.05$), or perceived to be striking or important, or have a positive direction of effect. Results may be unproven, but early results are promising.<br>Neutral: (a) no improvement, results were not statistically significant ($P >0.05$), or perceived to be unimportant, or showed null effect; (b) a mixture of positive and negative outcomes reported.<br>Negative: patients were worse off, current treatment is better than experimental treatment.<br>Unknown: Not known at this point in trial, not enough data, not explicitly reported.
4. Statements on Further Clinical Research	Yes: authors advocate for continued study in this area.<br>No: authors advocate against continued study in this area.<br>Unknown: no explicit statement on continuation or the trial is still in progress.
